# Finite-Element Computer Modeling of Spatial Displacement of the Pterygoid Venous Plexus During Mandibular Movements

**DOI:** 10.3390/jpm16050258

**Published:** 2026-05-12

**Authors:** Hadi Darawsheh, Dmitry Leonov, Sergey Dydykin, Beatrice Volel, Ellina Velichko, Irina Usmanova, Irina Lakman, Anzhela Brago, Seyedamirhossein Hosseini, Evgeniy Sosnin, Yuriy Vasil’ev

**Affiliations:** 1 First Moscow State Medical University (Sechenov University), N.V. Sklifosovsky Institute of Clinical Medicine, 119435 Moscow, Russia; daraushe_kh_m@staff.sechenov.ru (H.D.); leonov_d_s@staff.sechenov.ru (D.L.); dydykin_s_s@staff.sechenov.ru (S.D.); beatrice.volel@gmail.com (B.V.); amirhosseini148@yahoo.com (S.H.); sos.ev55@mail.ru (E.S.); 2 First Moscow State Medical University (Sechenov University), Institute of Digital Biodesign and Artificial Intelligence in Medicine, 119435 Moscow, Russia; velichko_e_v@staff.sechenov.ru; 3 Therapeutic Dentistry Department, BSMU, 450000 Ufa, Russia; irinausma@mail.ru; 4 Laboratory of Research of Socio-Economic Problems of Regions, Ufa University of Science and Technology, 450076 Ufa, Russia; lackmania@mail.ru; 5 Medical Institute, RUDN University, 119311 Moscow, Russia; anzhela_bogdan@mail.ru; 6 MIREA—Russian Technological University, Lomonosov Institute of Fine Chemical Technologies, Department of Cellular Systems Engineering, 125993 Moscow, Russia

**Keywords:** pterygoid venous plexus, inferior alveolar nerve block, finite-element method, safety

## Abstract

The safety of mandibular anesthesia is directly dependent on a precise understanding of the spatial relationships in the pterygomandibular space, particularly the risk of injury to the highly vascularized pterygoid venous plexus (PVP). In vivo studies of PVP displacement during mandibular movements face significant technical challenges. **Objective**: The study aims to study the spatial displacements of the pterygoid venous plexus during various physiological positions of the mandible using computer modeling with the finite-element method (FEM). **Materials and Methods**: A three-dimensional finite-element model was developed based on computed tomography data and the BodyParts3D anatomical atlas. The model included the bony structures of the skull, mandible, temporomandibular joint, masticatory muscles, and blood vessels. Simulations were performed for vertical displacements of the jaw at 15, 25, and 35 mm, as well as horizontal displacements of 5 mm to the left and right. **Results**: It was found that the magnitude of PVP displacement is proportional to the degree of mouth opening. The maximum total displacement (1.24 mm) was recorded at a 35 mm opening along the “posterior–medial–inferior” vector. Lateral excursions revealed asymmetry: displacement to the right caused plexus movement posteriorly, medially, and inferiorly (0.66 mm), while displacement to the left resulted in movement anteriorly, laterally, and superiorly (0.64 mm). **Conclusions**: This study demonstrates the significant mobility of the pterygoid venous plexus, which depends on the direction and amplitude of mandibular movements. The obtained data have important practical implications for planning regional anesthesia and minimizing the risk of iatrogenic complications. From a biomechanical perspective, maximum mouth opening produces the greatest displacement of the PVP, which may hypothetically reduce the risk of vascular puncture. Clinical studies are required to confirm this.

## 1. Introduction

Inferior alveolar nerve block or mandibular regional anesthesia is the most common method of injection anesthesia in dental practice. This technique, performed with appropriate precautions, is considered a relatively simple and effective procedure, involving the insertion of a needle into the pterygomandibular space to inject a local anesthetic in the immediate vicinity of the nerve. Successful anesthesia provides analgesia of the lower lip, gums, and teeth of the corresponding half of the lower jaw to the midline. To increase the effectiveness of mandibular nerve anesthesia, various techniques have been described in the literature, including the Clark and Holmes technique, the Gow-Gates technique, and the Sargenti technique. These approaches differ in parameters such as the direction of needle insertion, the depth of injection, and the zone of diffusion of the anesthetic, as well as in the degree of technical complexity of the performance. The Gow-Gates method is highly effective and involves inserting a needle parallel to an imaginary line connecting the tragus of the ear and the corner of the mouth. The needle is advanced to contact with the neck of the condylar process, while contact with the bone usually occurs at a depth of 25 mm [1,2,3].

The proximity of vessels, in particular the pterygoid venous plexus (PVP), to the head of the condylar process (MO) can provoke many local and general complications due to damage to the integrity of arteries and veins [4,5,6,7].

The pterygoid plexus is a complex of veins located in the subtemporal fossa of the skull, with extensive connections to the surrounding veins and anatomical structures. The pterygoid plexus borders the medial and lateral pterygoid muscles along with the temporal muscle, while the maxillary artery is surrounded by a plexus. As a result, the tributaries of the venous plexus are parallel to the branches of the maxillary artery. A clinically significant property of the pterygoid plexus is its communication with the cavernous sinus, inferior ophthalmic vein, and facial vein [8,9].

Conducting in vivo anatomical examinations has several technical challenges, and their conduct in humans is associated with fundamental ethical requirements, including respect for the donor, guarantees of confidentiality, and minimization of any potential harm [10]. The use of computer modeling techniques, such as finite-element analysis (FEA), circumvents many of these ethical dilemmas associated with invasive procedures on living people or cadaveric material. However, this raises new ethical questions related to the origin and use of the raw data, the validity of the models, and their potential clinical applications [11]. In this study, ethical aspects were considered at all stages: the use of computed tomography data was approved by the local ethics committee with the informed consent of the donor, and the model itself was strictly anonymized. Considering that the goal of the work is to improve the safety of medical interventions, this study was conducted in strict accordance with the ethical principles of respect for the person, beneficence, and justice.

The aim of the study was to study the spatial displacements of the pterygoid venous plexus at different positions of the mandible using computer modeling and the finite-element method.

## 2. Materials and Methods

### 2.1. Study Design

This study was approved by the Local Ethics Committee of the I.M. Sechenov First Moscow State Medical University (No: 24-24, approval date: 3 October 2024). The dependence of PVP movements on different mandibular positions was studied using computer modeling (Rassvet LLC, Moscow, Russia) and in vivo imaging. Modeling made it possible to obtain quantitative data on the displacement of the plexus at different positions of the mandible, namely.

- Vertical jaw displacement by 35 mm (maximum mouth opening);
- 25 mm vertical offset;
- 15 mm vertical offset;
- Horizontal displacement of 5 mm in both directions.

Measurements were performed for all positions in the right vascular complex.

### 2.2. Computed Tomography (CT) Data and Modeling

**Figure 1:**
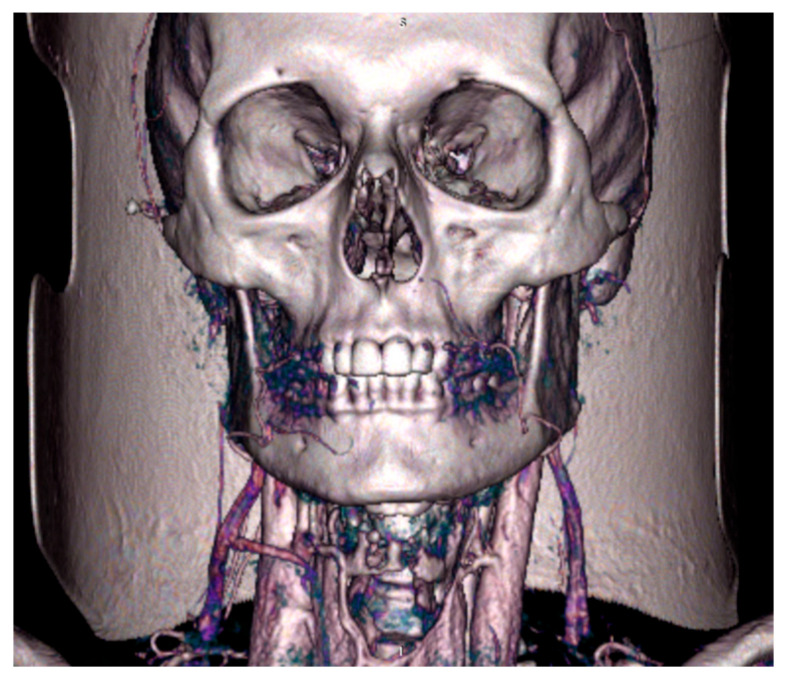
Three-dimensional reconstruction of multiphasic computed tomography angiography of the head and neck. The reconstruction was performed using a Canon Aquilion ONE dynamic 640-slice CT scanner. The image shows the bony structures, major arteries (external carotid artery and its branches), and the pterygoid venous plexus (PVP) before segmentation.

Multiphasic angiography of the head and neck of patient C (woman, 48 years old) without detected pathologies in the vascular system of the head and neck was performed on a Canon Aquilion ONE tomograph, dynamic 640-slice CT (Figure 1).

#### Angiography of the Head and Neck

From the data obtained, the surface of the voxel model was transformed into a polygonal shell (STL model) using the triangulation algorithm. Next, the obtained data was transported to CAE with the subsequent creation of a solid-state NURBS model.

**Figure 2:**
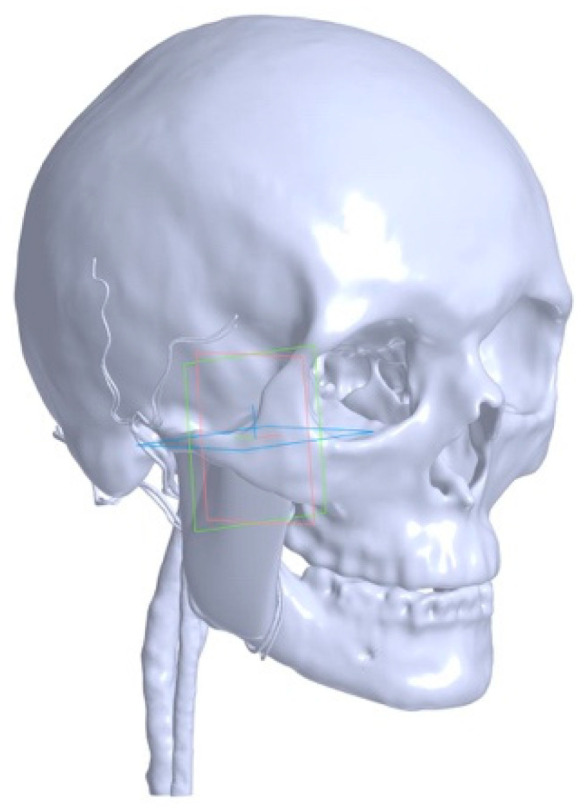
Solid-state NURBS model derived from CT data and the BodyParts3D anatomical atlas. The model includes the skull, mandible, temporomandibular joint (TMJ), masticatory muscles (masseter, medial pterygoid, lateral pterygoid), external carotid artery, internal jugular vein, and the pterygoid venous plexus (PVP).

The model includes the mandible, skull, temporomandibular joint, external carotid artery and its branches, pterygoid venous plexus, internal jugular vein, masseter muscle, medial pterygoid muscle, and lateral pterygoid muscle, modeled according to human anatomy based on an open database of anatomical structures, BodyParts3D (Figure 2).

### 2.3. Assumptions

The simulation was carried out considering the following conditions:

- A geometric model of the base of the skull, jaw, arteries of the head and neck was built according to the CT scan of patient C; Muscles were modeled using the BodyParts3D database.
- The geometry of the model was conventionally assumed to be symmetrical with respect to the middle line.
- The materials of the structures are homogeneous, elastic, and isotropic.
- Rigidly fixed geometry at the base of the skull. Displacements are set according to the physiological movements of the jaw.
- The calculation is carried out considering the main loads. Loads from muscle tension and contraction are considered the main loads.

These assumptions are widely used in biomechanical modeling of the maxillofacial region [12,13,14]. The physical and mechanical properties of the materials of the parts are accepted according to the calculation standards at 36 °C. Nominal permissible stresses are not determined (Table 1). The values of the physical and mechanical properties of the materials of the main parts are given in Table 1, where Rm is the minimum value of tensile resistance; p is density; v is Poisson’s ratio; and E is the modulus of elasticity of the first kind (Young’s modulus).

### 2.4. Finite-Element Model

The computational basis of the mechanics of a deformable body (pterygoid venous plexus) is the use of the finite-element method, the essence of which is to discretize an object into a finite set of elements. Finite elements vary in shape and size. As a result of sampling, a mesh is created from the boundaries of the elements, the intersection of which is formed by nodes. The set of all finite elements is a finite-element model of the body undergoing deformation.

The finite-element mesh was generated using second-order tetrahedral elements (10 nodes per element). A mesh convergence study was performed by successively refining the mesh until the change in the maximum total displacement of the PVP between two consecutive refinements fell below 2% [16]. The final mesh consisted of 487,000 elements and 712,000 nodes.

**Figure 3:**
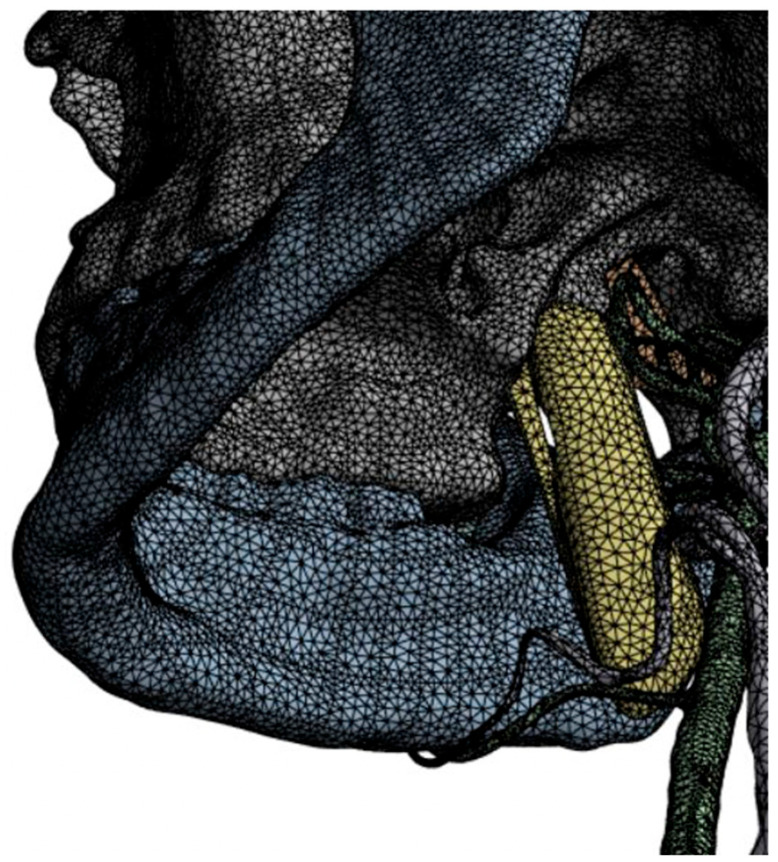
Finite-element mesh of the craniomandibular system.

The calculation was performed using the APM Multiphysics 21 multi-purpose universal complex for engineering analysis of structures and engineering methods according to generally accepted methods. APM software (v10.3.0, 2025) products are certified in accordance with the standards and requirements of the Federal Service for Environmental, Technological and Nuclear Supervision (Rostekhnadzor) and the Federal Budgetary Institution “Scientific and Technical Center for Nuclear and Radiation Safety” (FBU “STC NRS”), registration number 488, dated 19 December 2019 (valid until 19 December 2029). The finite-element model is made in accordance with the requirements of ISBN 5-9810-103-4 (Figure 3).

Boundary conditions: The skull base was rigidly fixed in all degrees of freedom (zero displacements in X, Y, Z directions). The temporomandibular joint (TMJ) was modeled as a frictionless contact between the condylar head and the articular disk, following the approach validated for jaw biomechanics [15,17].

Muscle load implementation: Muscle forces (masseter, medial pterygoid, lateral pterygoid, and temporalis) were applied as distributed loads over their physiological attachment areas, rather than concentrated forces, to avoid local stress artifacts [18]. Force magnitudes were derived from the physiological cross-sectional areas of each muscle, with activation levels corresponding to the specific jaw position (mouth opening of 15, 25, 35 mm; lateral excursions of 5 mm), based on electromyographic data from the literature [15].

Sensitivity analysis: To assess the robustness of the results, a sensitivity analysis was performed by varying the elastic modulus of soft tissues (muscles, vessels, ligaments) by ±20% [19]. The direction of PVP displacement remained unchanged across all variations, and the magnitude of total displacement changed by less than 12%, confirming the stability of the model predictions [20].

Verification and validation: The model was verified through mesh convergence and sensitivity analyses. Qualitative validation was performed by comparing the displacement patterns with known anatomical relationships [21].

The task of the calculation is to numerically determine the displacements of vessels along the abscissa, ordinate, and applicate axes in a rectangular Cartesian coordinate system resulting from the movements of the jaw. The results when the jaw is opened at 35 mm are presented using the “Probe” command, which allows for numerical display of the result at a specific point in a rectangular Cartesian coordinate system. Subsequent results are presented as result maps using a color chart that corresponds to the diagram on the result scale.

## 3. Results

**Figure 4:**
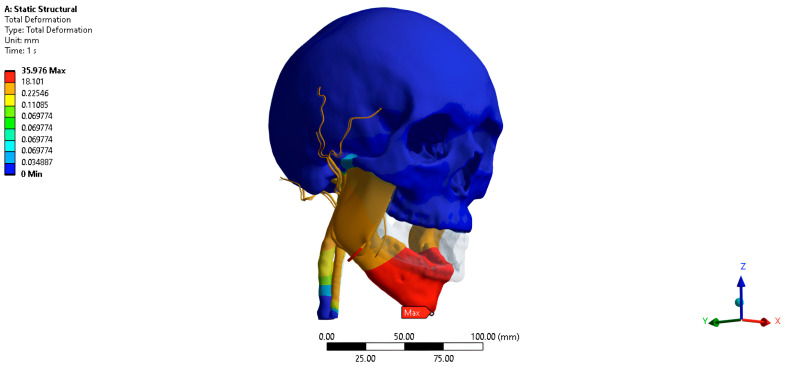
Simulated displacement of the pterygoid venous plexus at maximum mouth opening (35 mm). The color map shows total displacement magnitude (in mm) ranging from blue (no displacement) to red (maximum displacement).

The total movement of the PVP when opening the jaw by 35 mm is 1.24 mm posteriorly, medially, and inferiorly (Figure 4). The movement of the PVP along the abscissa axis when opening the jaw by 35 mm is −0.53 mm. Movement of the PVP along the ordinate axis when the jaw is opened by 35 mm is −1.23 mm. Movement of the PVP along the axis of the application when opening the jaw by 35 mm is −0.86 mm.

**Figure 5:**
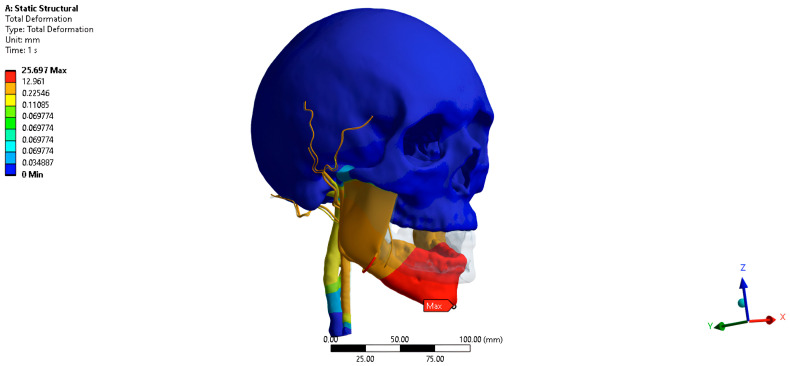
Finite-element model at 25 mm of jaw opening.

The total movement of the PVP when opening the jaw by 25 mm is 0.97 mm posteriorly, medially, and inferiorly (Figure 5).

The movement of the PVP along the abscissa axis when opening the jaw by 25 mm is −0.42 mm.

Movement of the PVP along the ordinate axis when the jaw is opened by 25 mm is −0.64 mm.

Movement of the PVP along the axis of the application when the jaw is opened by 25 mm is −0.53 mm.

**Figure 6:**
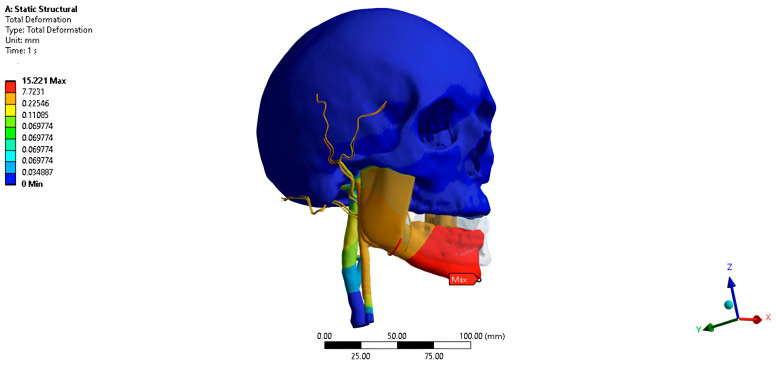
Finite-element model at 15 mm of jaw opening.

The total movement of the PVP when opening the jaw by 15 mm is 0.56 mm posteriorly, medially, and inferiorly (Figure 6). The movement of the PVP along the abscissa axis when opening the jaw by 15 mm is −0.24 mm. Movement of the PVP along the ordinate axis when the jaw is opened by 15 mm is −0.33 mm.

Movement of the PVP along the axis of the application when opening the jaw by 15 mm is −0.31 mm.

The maximum reduced displacements of the PVP along the aggregate of axes increased proportionally to the amplitude of jaw opening. When opening the mouth by 15 mm, the total displacement was 0.56 mm; at 25 mm, it was 0.97 mm, and at 35 mm—1.24 mm.

Along the coordinate axes, the movements were distributed as follows: *X*-axis (lateral-medial direction): from −0.24 mm at 15 mm opening to −0.53 mm at 35 mm; *Y*-axis (anterior–posterior): from −0.33 mm at 15 mm opening to −0.23 mm at 35 mm opening, indicating retroposition of the PVP with increased mouth opening; *Z* axis (vertical axis): from −0.31 mm at 15 mm to −0.86 mm at 35 mm, which reflects the lowering of the PVP when the lower jaw is opened.

**Figure 7:**
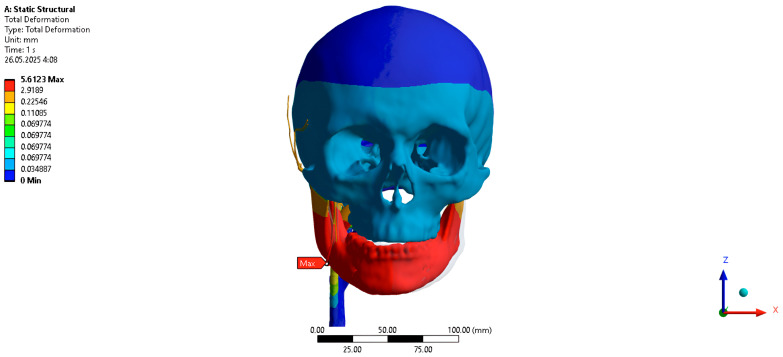
Finite-element model with a 5 mm rightward lateral shift of the jaw.

The total PVP movement when the jaw is shifted 5 mm to the right with the mouth closed is 0.66 mm posteriorly, medially, and inferiorly (Figure 7).

PVP movement along the abscissa axis when the jaw is shifted 5 mm to the right is −0.52 mm, along the ordinate axis when the jaw is shifted 5 mm to the right is −0.39 mm, along the axis of the appliqué when the jaw is shifted 5 mm to the right is −0.15 mm.

**Figure 8:**
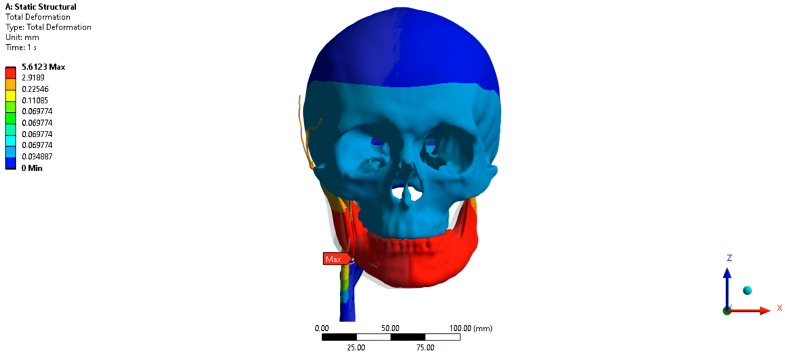
Finite-element model with a 5 mm leftward lateral shift of the jaw.

The total PVP movement when the jaw is shifted 5 mm to the left is 0.64 mm anteriorly, outward, upwards (Figure 8). The PVP movement along the abscissa axis when the jaw is shifted 5 mm to the left is 0.48 mm, along the ordinate axis when the jaw is shifted 5 mm to the left is 0.40 mm, along the axis of the applicate when the jaw is shifted 5 mm to the left is 0.11 mm.

With lateral movement of the jaw by 5 mm to the left, the total PVP displacement was 0.64 mm, and with a shift to the right, it was 0.66 mm. Summary data on PVP movements at different mandibular positions are presented in Table 2.

Analysis of the computer simulation data suggests that the degree of spatial displacement of PVP is a key factor in determining the safety of the injection. The greatest total displacement of the structure is observed at the maximum opening of the mouth by 35 mm (1.24 mm), which indicates a significant distance of the plexus from its neutral position. Therefore, this position of the mandible can be considered as the most preferable for minimizing the risk of puncture, since the zone of potential damage is displaced to the greatest extent.

Thus, to ensure safe manipulation, it is advisable to use the position of maximum mouth opening, at which PVP demonstrates the greatest displacement along the vector “posterior–inward–downward”.

## 4. Discussion

Our finite-element study provided the first quantitative data on the displacement of the pterygoid venous plexus (PVP) in various physiological positions of the mandible. PVP displacement is proportional to the degree of mouth opening, reaching a maximum total displacement of 1.24 mm at an opening of 35 mm along the posteromedial–inferior vector. Lateral displacements of 5 mm result in asymmetric displacement: a rightward displacement displaces the PVP posteromedially and inferiorly (0.66 mm), while a leftward displacement displaces it anterolaterally and superiorly (0.64 mm).

These results extend existing anatomical knowledge. The PVP is known to be tightly associated with the lateral pterygoid muscle, which acts as a venous pump during mandibular movements [22,23]. However, previous anatomical descriptions were static and could not provide quantitative displacement values. Our findings confirm that the PVP is not a fixed structure but moves in a direction- and amplitude-dependent manner, which has direct implications for the safety of mandibular nerve blocks.

From a biomechanical modeling perspective, our results are consistent with previous data on the spatial structure. Darawsheh et al. (2023) [13] used a finite-element method to analyze the optimal mandibular position for the mandibular nerve block (IANB) and found that neurovascular bundle displacement was sensitive to jaw position. Similarly, Toro-Ibakache et al. (2016) [24] showed that the direction of soft tissue displacement in the masticatory system was relatively little related to moderate changes in muscle force magnitude. This confirms the directional robustness of our results, even though our muscle loads were derived from literature data rather than individual measurements. Our sensitivity analysis (±20% change in elastic modulus resulting in a change in total displacement of less than 12%) further confirms this stability.

The clinical significance of the obtained displacement values, particularly 1.24 mm at maximum mouth opening, should be interpreted in relation to the incidence of complications during mandibular anesthesia. The distance from the needle tip to the pterygohomandibular space in the neutral jaw position is not precisely known, but anatomical studies of the pterygohomandibular space have shown significant individual variability. Moreover, the distances between key structures vary by several millimeters [25,26]. A displacement of 1.24 mm is comparable to natural anatomical variation and may contribute to an increase in the “safe zone” between the needle trajectory and the venous plexus. Furthermore, the incidence of unintentional intravascular injections during BANN, according to the literature, ranges from 0.08% to 22% depending on the technique [27,28]. Techniques that require wide mouth opening, such as the Gow-Gates technique, are associated with lower rates of positive aspiration [3]. Our results provide a biomechanical explanation for this clinical observation: maximum mouth opening displaces the PVS posteriorly and inferiorly, away from the typical injection site, thereby reducing the likelihood of accidental venipuncture.

The asymmetrical nature of the displacement during lateral movements to the right and left is an unexpected but important finding. Although our model assumed geometric symmetry, the loading conditions (the direction of muscle forces and joint constraints) resulted in different vectors. This suggests that even in a symmetrical model, the biomechanical response to lateral jaw movements is inherently asymmetric. In clinical practice, this may indicate that the choice of side for anesthesia may influence the risk of injury to the pterygoid venous plexus. Several methodological limitations must be acknowledged. Our model was based on CT data from a single subject (a 48-year-old woman), and the masticatory muscles were taken from an anatomical atlas rather than being segmented from the same individual. Therefore, interindividual anatomical variability is not considered.

In this study, all tissues were assumed to be homogeneous, isotropic, and linearly elastic, whereas biological tissues exhibit anisotropic, nonlinear, and viscoelastic behavior. These simplifications are standard in finite-element analysis of the maxillofacial region [12,13] and are acceptable for comparative analysis of displacement patterns, but they affect the absolute displacement magnitudes. We did not perform direct muscle measurements, as the data were obtained from literature values of physiological cross-sectional areas and electromyographic activation levels [15]. Sensitivity analysis confirmed that the direction of displacement remained constant and the magnitude varied by less than 12%, indicating that the main conclusions are robust to uncertainties in the material parameters. We did not conduct direct in vivo validation (e.g., dynamic MRI, 4D flow MRI, or cadaveric testing), which is a recognized challenge for deep craniofacial venous structures [21].

Therefore, absolute displacement values should be considered approximate and suggestive rather than clinically validated. We did not conduct direct quantitative validation using dynamic imaging (e.g., cine MRI or 4D flow MRI) or cadaveric measurements.

In summary, the absolute displacement values presented here should be considered model-dependent estimates that require confirmation using independent methods. Personalized FEM models based on large patient cohorts are needed to assess interindividual variability. Dynamic imaging techniques, such as cine MRI or 4D flow MRI, could potentially validate our displacement models in living subjects. Based on the difference in positive aspiration rates between the Gow-Gates technique and traditional methods (approximately 8–10%) and assuming a linear relationship, the 1.24 mm displacement observed in our model could correspond to a clinically significant reduction in the risk of vascular puncture of a similar magnitude. Anatomical studies of the pterygomandibular space show that the distance from the tip of a conventional mandibular nerve block needle (inserted to a standard depth of 20–25 mm) to the nearest wall of the pterygoid venous plexus ranges from 2.0 to 4.5 mm, depending on individual anatomy and needle trajectory [26]. Thus, a 1.24 mm offset represents 25–60% of the available safety margin. From a biomechanical perspective, this reduction in the likelihood of accidental venipuncture can be estimated using a simplified geometric model: if the pterygomandibular space is modeled as a cylindrical target with a diameter of 3 mm, a 1.24 mm offset perpendicular to the needle trajectory reduces the cross-sectional overlap area by approximately 40–50%. This suggests that maximum mouth opening can reduce the risk of intravascular injection by nearly half compared to a neutral jaw position.

These estimates, although theoretical, provide quantitative justification for the clinical preference for wide mouth opening and should be validated in prospective studies. In conclusion, this study demonstrates that the PVP undergoes measurable and directionally dependent displacement during mandibular movements, with maximum mouth opening producing the greatest displacement from the neutral position. Although absolute values remain model-dependent, the proportional relationship and directional patterns are robust and provide biomechanical justification for considering wide mouth opening as a potentially safer position for mandibular anesthesia, pending clinical validation.

## 5. Conclusions

The study demonstrates a clear dependence of the spatial position of the PVP on the direction and amplitude of mandibular movements. It has been established that with vertical displacements, the magnitude of the displacement of the plexus along the posterior–medial–inferior vector directly depends on the degree of mouth opening, reaching a maximum at 35 mm. This indicates a complex and asymmetric biomechanical interaction in the temporomandibular joint. Thus, the PVP is a highly mobile structure, and its displacements during lateral movements of the jaw have a multidirectional character.

## Figures and Tables

**Table 1:** Physical and mechanical properties of materials.

Detail	Material	Parameter		Temperature, °C
		Designation	Unit	36
Skull, jaw bone	Bone	R_m_	MPa	Not Specified
		p	Kg/m^3^	1900.0
		v	-	0.23
		E	MPa	14,000.0
Muscle	Muscle	R_m_	MPa	Not Specified
		p	Kg/m^3^	1090.0
		v	-	0.3
		E	MPa	0.3
Ligaments	Ligament	R_m_	MPa	Not Specified
		p	Kg/m^3^	-
		v	-	0.35
		E	MPa	90
Nerves	Nerve	R_m_	MPa	Not Specified
		p	Kg/m^3^	-
		v	-	0.49
		E	MPa	3
TMJ	TMJ	R_m_	MPa	Not Specified
		p	Kg/m^3^	-
		v	-	0.49
		E	MPa	20
Arteries	Arteries	R_m_	MPa	Not Specified
		p	Kg/m^3^	-
		v	-	0.49
		E	MPa	0.7

Parameters marked as ‘Not Specified’ (e.g., Rm) are not required for linear elastic FEM calculations and do not affect the displacement results; they are included for completeness. Material properties (density ρ, Poisson’s ratio ν, Young’s modulus E) were taken from [12,13,15] for bone, muscle, ligaments, nerves, TMJ, and arteries.

**Table 2:** Summary of PVP movements at different mandibular positions.

Jaw Position	Total, mm	X, mm (Lateral-Medial Direction)	Y, mm (Anterior–Posterior Direction)	Z, mm (Vertical Axis)
Opening 35 mm	1.24	−0.53	−1.23	−0.86
Opening 25 mm	0.97	−0.42	−0.64	−0.53
Opening 15 mm	0.56	−0.24	−0.33	−0.31
Rightward shift 5 mm	0.66	−0.52	−0.39	−0.15
Leftward shift 5 mm	0.64	0.48	0.4	0.11

## Data Availability

The data presented in this study are available on request from the corresponding author according to Russian law.
